# Development and Application of Nanoparticles in Biomedical Imaging

**DOI:** 10.1155/2018/1403826

**Published:** 2018-04-01

**Authors:** Paulo H. Rosado-de-Castro, María del Puerto Morales, Pedro M. Pimentel-Coelho, Rosalia Mendez-Otero, Fernando Herranz

**Affiliations:** ^1^Instituto de Ciências Biomédicas, Universidade Federal do Rio de Janeiro, Av. Carlos Chagas Filho 373, 21941-590 Rio de Janeiro, RJ, Brazil; ^2^D'Or Institute for Research and Education, 22281-100 Rio de Janeiro, RJ, Brazil; ^3^Departamento de Energía, Medioambiente y Salud, Instituto de Ciencia de Materiales de Madrid, CSIC, Sor Juana Inés de la Cruz 3, Cantoblanco, 28049 Madrid, Spain; ^4^Instituto de Biofísica Carlos Chagas Filho, Universidade Federal do Rio de Janeiro, Av. Carlos Chagas Filho 373, 21941-590 Rio de Janeiro, RJ, Brazil; ^5^CIBER Enfermedades Respiratorias (CIBERES) and Fundación Centro Nacional de Investigaciones Cardiovasculares (CNIC), Melchor Fernández-Almagro 3, 28029 Madrid, Spain

The combination of the size-dependent properties of nanomaterials with the noninvasive characterisation in molecular imaging is a powerful combination that is being successfully applied across disciplines. In the past ten years, we have witnessed the development of, literally, hundreds of nanoparticle-based probes for molecular imaging. All major imaging techniques have been enhanced by the use of nanoparticles, particularly magnetic resonance imaging (MRI), positron emission tomography (PET), and optical imaging. The use of iron oxide nanoparticles for T_1_-weighted and/or T_2_-weighted MRI, the design of radioisotope chelator-free particles for PET, and new developments in fluorescent nanoparticles (carbon dots and upconverting nanoparticles) are important milestones in the field. There are two key features in nanoparticle-based probes which are seldom found in traditional imaging probes: multimodality and multifunctionality. The use of, at least, two complementary imaging techniques (multimodality) like PET/MRI or MRI/Fluorescence and the possibility of incorporating several vectors on the surface and/or drugs (multifunctionality) expand the use of these probes. Furthermore, the properties of some nanoparticles can be used to create new imaging techniques, for example, the superparamagnetism of iron oxide nanoparticles for magnetic particle imaging.

In this issue, we have aimed to provide a platform for high-quality contributions on nanoparticles application to molecular imaging. Original papers and review articles focusing on the latest application of nanoparticle-based imaging probes were submitted. The topics treated include the application of iron oxides for MRI, for PET/MRI, and for drug delivery; new synthesis approaches to obtain magnetic nanoparticles-based contrast agents; polymeric nanoparticles for ultrasound imaging; new computed tomography (CT) contrast agents; and quantum dots for multiplex optical imaging. We received a total of 16 submissions, and after two rounds of rigorous review, 9 papers were accepted for publications in this special issue.

